# Enhancing the Maturation of Human Pluripotent Stem Cell-Derived Cardiomyocytes with an n-Type Organic Semiconductor Coating

**DOI:** 10.1021/acsami.3c18919

**Published:** 2024-04-15

**Authors:** Gustavo Ramirez-Calderon, Abdulelah Saleh, Tania Cecilia Hidalgo Castillo, Victor Druet, Bayan Almarhoon, Latifah Almulla, Antonio Adamo, Sahika Inal

**Affiliations:** ‡ Laboratory of Stem Cells and Diseases, Biological and Environmental Science and Engineering Division, King Abdullah University of Science and Technology (KAUST), Thuwal 23955-6900, Saudi Arabia; § Organic Bioelectronics Laboratory, Biological and Environmental Science and Engineering Division, KAUST, Thuwal 23955-6900, Saudi Arabia

**Keywords:** conjugated polymer, n-type, stimulation, qPCR, organic bioelectronics, cardiomyocyte, pluripotent, stem cell

## Abstract

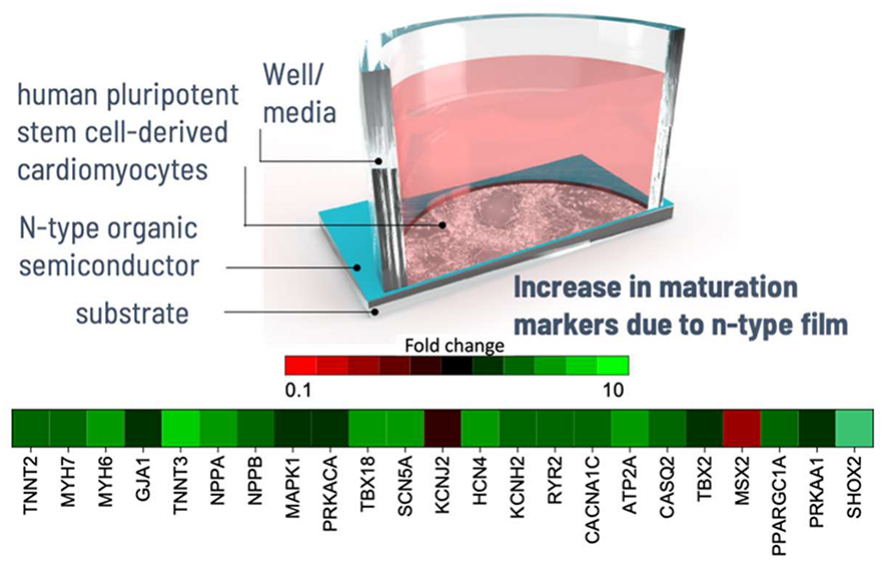

Human pluripotent stem cell-derived cardiomyocytes (hPSC-CMs) are a promising cell source for cardiac regenerative medicine and *in vitro* modeling. However, hPSC-CMs exhibit immature structural and functional properties compared with adult cardiomyocytes. Various electrical, mechanical, and biochemical cues have been applied to enhance hPSC-CM maturation but with limited success. In this work, we investigated the potential application of the semiconducting polymer poly{[N,N′-bis(2-octyldodecyl)-naphthalene-1,4,5,8-bis(dicarboximide)-2,6-diyl]-alt-5,5′-(2,2′-bithiophene)} (P(NDI2OD-T2)) as a light-sensitive material to stimulate hPSC-CMs optically. Our results indicated that P(NDI2OD-T2)-mediated photostimulation caused cell damage at irradiances applied long-term above 36 μW/mm^2^ and did not regulate cardiac monolayer beating (after maturation) at higher intensities applied in a transient fashion. However, we discovered that the cells grown on P(NDI2OD-T2)-coated substrates showed significantly enhanced expression of cardiomyocyte maturation markers in the absence of a light exposure stimulus. A combination of techniques, such as atomic force microscopy, scanning electron microscopy, and quartz crystal microbalance with dissipation monitoring, which we applied to investigate the interface of the cell with the n-type coating, revealed that P(NDI2OD-T2) impacted the nanostructure, adsorption, and viscoelasticity of the Matrigel coating used as a cell adhesion promoter matrix. This modified cellular microenvironment promoted the expression of cardiomyocyte maturation markers related to contraction, calcium handling, metabolism, and conduction. Overall, our findings demonstrate that conjugated polymers such as P(NDI2OD-T2) can be used as passive coatings to direct stem cell fate through interfacial engineering of cell growth substrates.

## Introduction

Human pluripotent stem cell-derived cardiomyocytes (hPSC-CMs) hold great potential as a cell source for myocardial regeneration,^1^ pharmacological studies,^2^ and disease modeling.^3^ However, they typically exhibit immature structural and functional properties compared to adult cardiomyocytes (CMs).^4^ Enhancing the maturation of hPSC-CMs is therefore critical for unlocking their full potential. Various approaches, including chemical, mechanical, and electrical stimuli, have been explored to promote the maturation of hPSC-CMs, albeit with moderate success.^5,6^ In particular, electrical stimulation has been proposed as a method to induce maturation of hPSC-CMs *in vitro.*(6,7) However, the design and implementation of electrical stimulation bioreactors face certain constraints,^8^ limiting the wide range adaptation of the technique. For example, the fact that electrodes must be physically inserted into the bioreactor and wired to external stimulation devices presents a configuration challenge for compatibility with conventional substrates and incubators.

Light-based pacing technologies offer a wireless alternative for delivering stimulation to cells.^9,10^ Materials such as graphene,^11^ silicon nanowires,^12^ gold nanoparticles,^13^ and conjugated polymers^14,15^ have shown the ability to optically pace CMs when exposed to light. The absorption of light by these materials generates effects such as photovoltage, heat increase, or reactive oxygen species (ROS) production, which can modulate CM pacing through various mechanisms.^16,17^ For example, one study has shown that when human induced pluripotent stem cell-derived CMs (hiPSC-CMs) were grown on a p-type (hole conducting) organic semiconductor (i.e., poly-3-hexylthiophene, P3HT), exposure to 550 nm light for 30 s resulted in an increase in their beating frequency.^14^ This frequency increase was attributed to local heating of the polymer coating upon light absorption, subsequently activating the cells. A similar phenomenon was observed with the p-type poly[2,1,3-benzothiadiazole-4,7-diyl[4,4-bis(2-ethylhexyl)–4H-cyclopenta[2,1-b:3,4-b’]dithiophene-2,6-diyl]] (PCPDTBT) films, which generated a temperature rise on their surface when illuminated.^15^ Alongside the heating effect upon light exposure, ROS were detected in the media near the PCPDTBT-cell interface. The authors inferred that the combination of temperature changes and ROS generation induced by polymer photoexcitation affected calcium handling proteins and, ultimately, CM beating frequency.^15^

These successful examples of light-based pacing technology can ideally be applied for long-term stimulation to induce maturation, similar to electrical stimulation. However, a significant challenge lies in determining safe light exposure conditions to prevent cell damage. Studies which observed changes in CM beating frequency have typically utilized high irradiances, approximately 6–20 mW/mm^2^, administered in short bursts under 30 s.. While these conditions prevent immediate cell death, they are not viable for prolonged pacing over days or weeks.^18^ The maximum exposure times and light intensities that maintain cell viability while providing effective, sustained pacing remain unclear. Understanding the interplay among light intensity, duration, and cell viability will be critical for translating these emerging optical pacing technologies for long-term applications.

Furthermore, in many of these light-sensitive pacing systems, the photoactive material serves as the substrate–coating onto which cells adhere, facilitating the transmission of local stimulation effects. However, there is some indication that these materials may also passively influence the cell properties. For example, P3HT exhibits no adverse effects on cells over multiple days of culture,^14^ while PCPDTBT not only demonstrates a lack of cytotoxicity but also enhances cell growth and function compared to inert substrates such as glass.^15^ Thus, organic semiconductor coatings may offer additional benefits by altering surface stiffness, topography, or physicochemical properties known to impact cell maturation.^19^ Understanding both the passive and active effects of these photoactive biomaterials will guide future optimization of CM tissue engineering constructs and optical pacing devices.

In this work, we investigated the potential of the n-type (electron transporting) polymer poly{[N,N′-bis(2-octyldodecyl)-naphthalene-1,4,5,8-bis(dicarboximide)-2,6-diyl]-*alt*-5,5′-(2,2′-bithiophene)} (referred to as P(NDI2OD-T2)) as a light-sensitive material for sustained optical pacing of hPSC-CMs. P(NDI2OD-T2) is a commercially available donor–acceptor type copolymer that has been extensively used in solar cells and field effect transistors due to its high electron mobility, light absorption, and stability in ambient conditions.^20^ We aimed to identify the minimum light intensity that could effectively pace hPSC-CMs without causing phototoxicity during prolonged exposures. Our results showed that even low-intensity light exposure resulted in cell damage with no instant benefits of light simulation. Interestingly, in the absence of photostimulation, simply culturing the hPSC-CMs on P(NDI2OD-T2)-coated substrates led to a significant enhancement in the expression of CM maturation markers. Using several techniques to probe the interface between the organic semiconductor film and the cells, we discovered differences in the cell adhesion promoter coating, i.e., Matrigel, on the P(NDI2OD-T2) surface compared with the control substrate. We hypothesize that the conformation of cell-adhesion proteins is influenced by the underlying substrate; for the P(NDI2OD-T2) case, this allowed us to change the fate of the cells. These results suggest that even when used passively, semiconducting polymers like P(NDI2OD-T2) can provide benefits beyond optical and electrical pacing by tuning the physicochemical properties of the cell growth environment that impact their function.

## Results and Discussion

### P(NDI2OD-T2) As a Substrate Coating for Cell Culture

**Figure 1:**
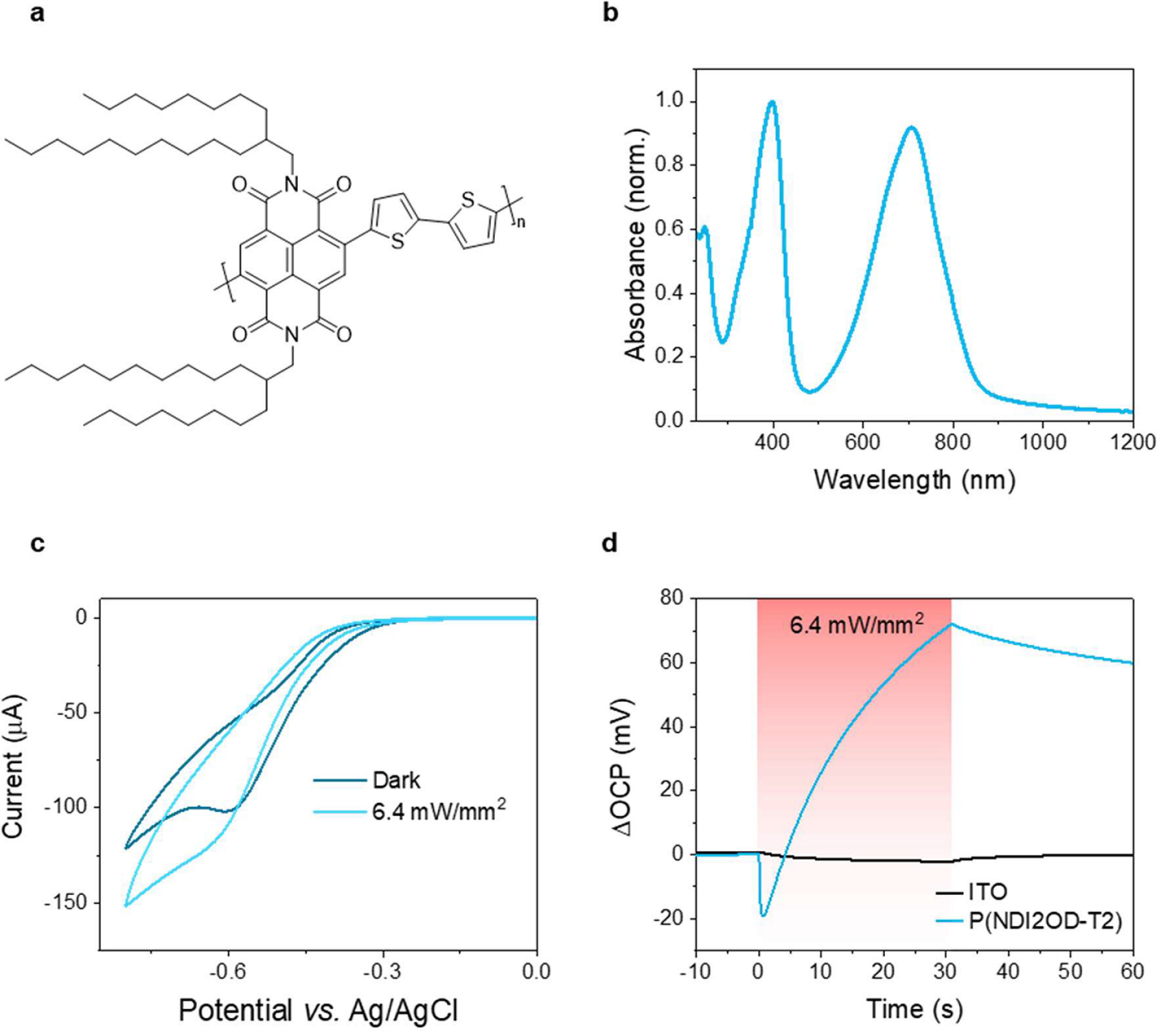
Optoelectronic properties of P(NDI2OD-T2) at the aqueous interface. (A) Chemical structure of P(NDI2OD-T2). (B) UV–vis spectrum of the P(NDI2OD-T2) film. (C) Cyclic voltammetry curves of the film recorded in cell media under dark conditions and during illumination with 660 nm red light with an irradiance of 6.4 mW/mm^2^. (D) Change in open circuit potential (OCP) of the underlying ITO substrate and that of the P(NDI2OD-T2)-coated ITO substrate during 30 s exposure to 660 nm red light.

P(NDI2OD-T2) is a photosensitive, n-type polymer with a backbone composed of the electron-deficient 2,6-dibromonaphthalene-1,4,5,8-tetracarboxylic diimide unit and the electron-rich bithiophene unit^20,21^ (see the chemical structure shown in Figure 1a). P(NDI2OD-T2) is renowned for its intriguing film morphology-transport performance relationships.^22,23^ Its transport properties have been observed to remain unaffected by elevated temperatures; however, the electron mobility decreases when the films are stored in the presence of indoor light due to interactions of the photoexcited species with oxygen.^20^ The thin film optical spectrum of P(NDI2OD-T2) on an indium tin oxide (ITO) substrate is shown in Figure 1b, revealing two main absorption peaks at 700 and 400 nm. For this study, we selected a 660 nm red light source to carry out all light stimulation experiments. This wavelength was chosen to avoid photobleaching of the film upon long-term exposure and ensure deep penetration into tissue,^24^ making it favorable for *in vivo* translation.

The cyclic voltammetry (CV) measurements performed in cell media revealed that the exposure to 660 nm light increased the reduction currents of P(NDI2OD-T2) obtained at reduction voltages above −0.5 V versus the Ag/AgCl reference electrode (Figure 1c). No differences in the CV curves recorded in the dark or light were observed at reduction potentials more positive than −0.3 V. However, in the absence of a bias, light exposure caused a shift in the open circuit potential (OCP) of the film (Figure 1d). P(NDI2OD-T2) electrodes exhibited an OCP change of *ca*. +70 mV during 30 s of exposure of light with an irradiance of 6.4 mW/mm^2^. As ITO is transparent to 660 nm, it showed no change in its OCP. Thus, the observed shift in OCP is solely attributable to P(NDI2OD-T2), suggesting the generation of free electron and hole pairs transporting at the electrolyte and ITO interfaces, respectively.^25−27^

**Figure 2:**
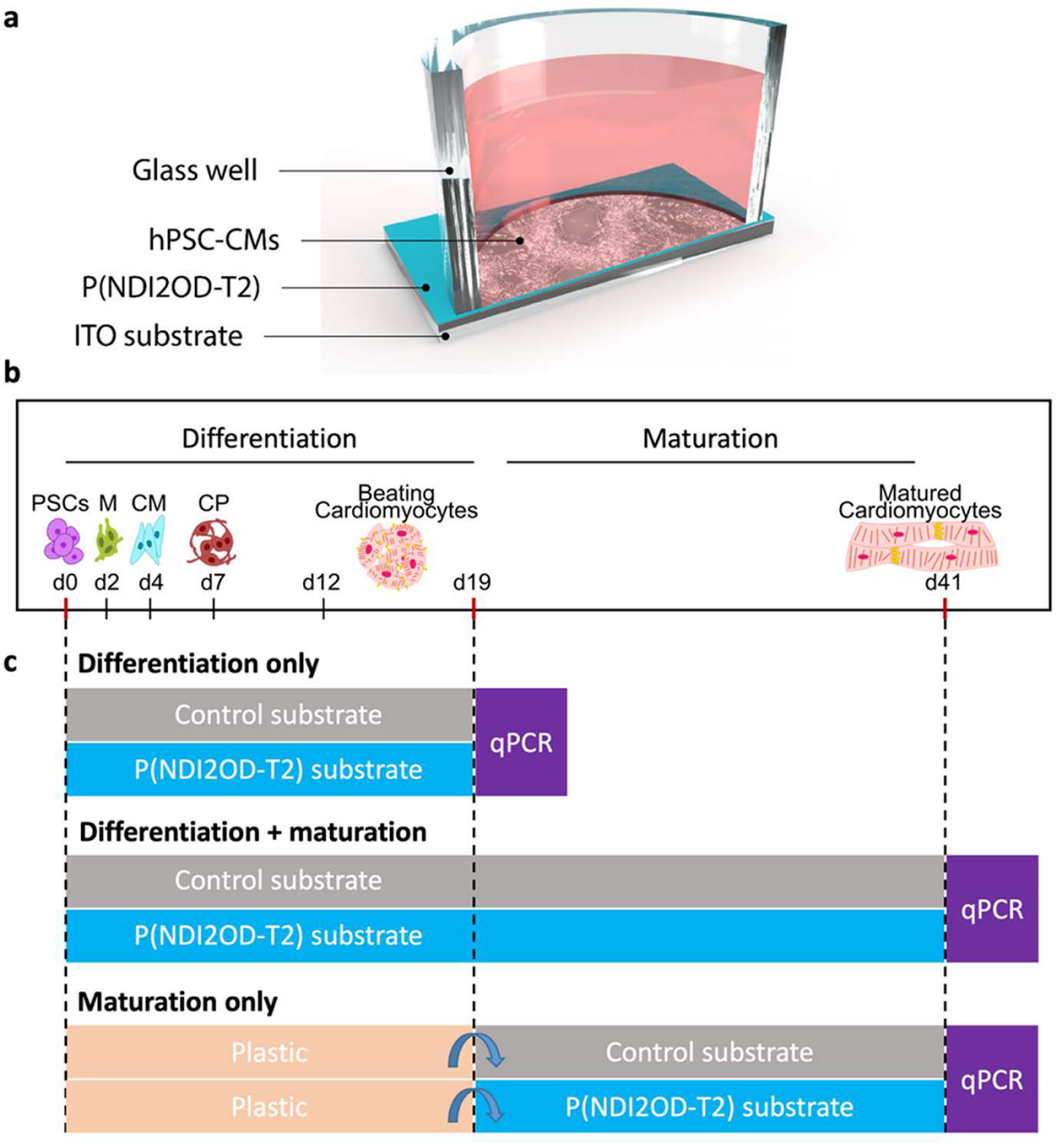
Experimental design. (A) Schematic of the bioelectronic device. Human pluripotent stem cell-derived cardiomyocytes (hPSC-CMs) are differentiated and cultured on P(NDI2OD-T2)-coated ITO substrates. (B) Differentiation and maturation timelines of hPSC-CMs. (C) Experiments were performed to test the effect of P(NDI2OD-T2) on the differentiation of hPSC-CMs (Differentiation only), the differentiation and maturation of hPSC-CMs (Differentiation + maturation), and the maturation of hPSC-CMs (Maturation only).

Given the observed light-induced shift in OCP and the previously demonstrated effect of photostimulation (i.e., photocapacitive) on depolarizing cell membranes,^28^ we sought to investigate whether P(NDI2OD-T2) might interfere with the functionality of hPSC-CMs. It is crucial to highlight that P(NDI2OD-T2) has not been previously utilized with stem cells, neither passively as a substrate nor actively for optical or electrical stimulation. Nevertheless, the physicochemical properties of substrates can influence the differentiation and maturation of stem cell-derived CMs.^29^ Therefore, before assessing P(NDI2OD-T2)’s optical pacing capability, we conducted experiments to evaluate its impact on hPSC adherence, differentiation, and maturation behavior in the absence of light stimulation. Figure 2a shows a schematic representation of our bioelectronic device comprising the hPSC-CMs grown on P(NDI2OD-T2)-coated ITO substrates. Control samples were cultured in the same manner but without the P(NDI2OD-T2) coating. Each device is a single-well substrate with an inner diameter of 14 mm and a height of 17.5 mm, with dimensions comparable to those of a standard 24-well plate well.

After 24 h of culture on both control and P(NDI2OD-T2) substrates, the cells displayed the anticipated hPSC colony morphology (not depicted). Subsequently, after 48 h on these substrates, we initiated our differentiation protocol, with day 0 (d0) marking the beginning, as illustrated in Figure 2b. From day 0 onward, the hPSCs were exposed to CM differentiation media, initiating mesoderm formation by day 2, progressing to cardiac mesoderm by day 4, and culminating in the onset of CM beating by day 12. By day 19 (d19), the cells had fully differentiated and entered the maturation stage, which continued until day 41 (d41).

To understand how P(NDI2OD-T2) affects hPSC-CM differentiation and maturation, we performed three separate experiments, each comprising triplicate samples to ensure statistical significance (Figure 2c). In the first experiment (“Differentiation only”), we examined the effect of P(NDI2OD-T2) on cell differentiation compared to the control. For this experiment, the cells were evaluated on d19 before they entered the maturation stage. In the second experiment (“Differentiation + maturation”), the hPSC-CMs were differentiated into cardiomyocytes on P(NDI2OD-T2) or control substrates for a total of 41 days to assess the combined effect of P(NDI2OD-T2) on both differentiation and maturation. To further delineate the timing of maturation onset, the third experiment (“Maturation only”) involved differentiating hPSCs into CMs on a standard tissue culture plate for the initial 19 days. Subsequently, hPSC-CMs were transferred onto P(NDI2OD-T2) and control substrates for the remaining 21 days of culture, representing the maturation phase exclusively.

**Figure 3:**
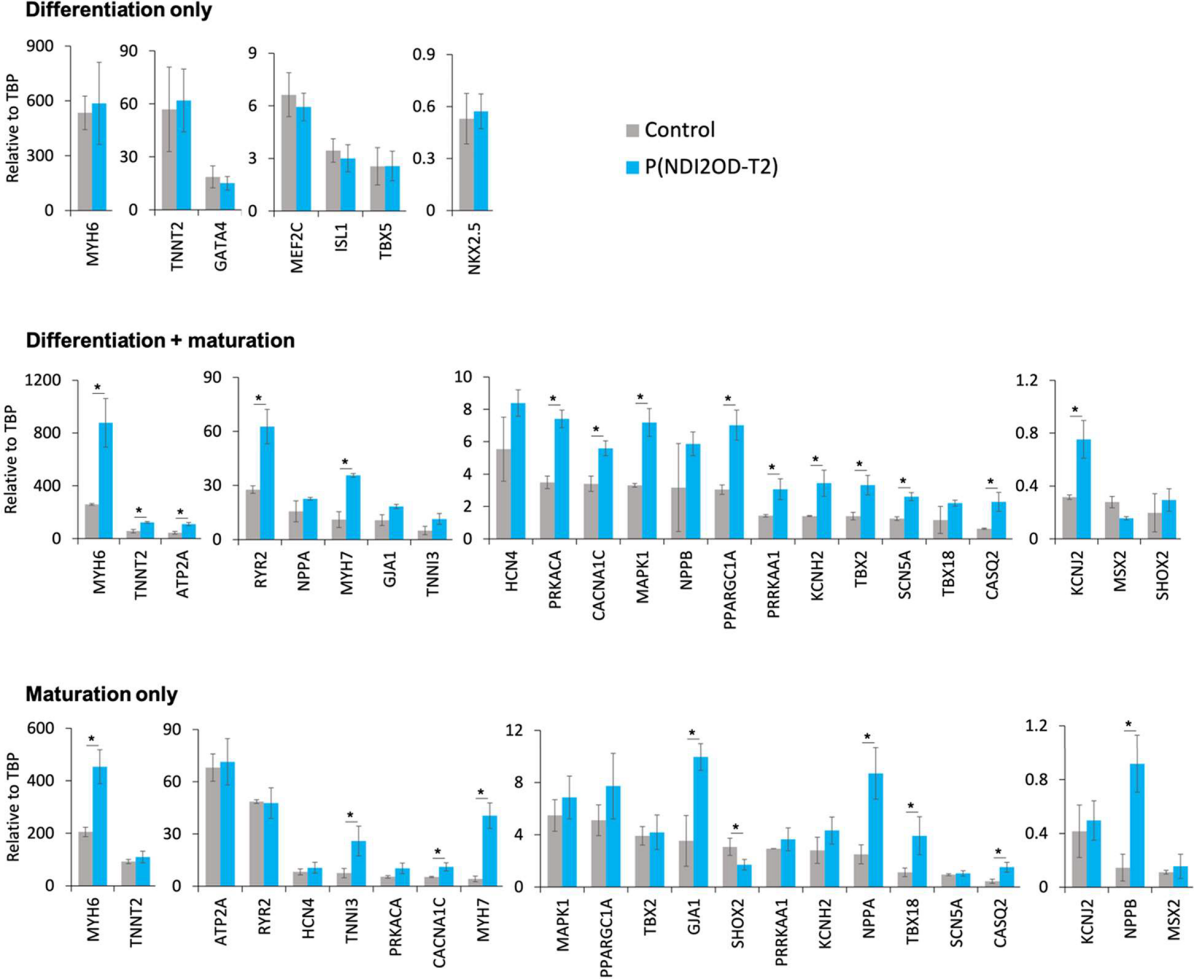
Gene expression analysis of hPSC-CMs. Differentiation and maturation marker expression derived from the qPCR experiments performed on *n* = 3 samples for each condition. * *p* < 0.05 (unpaired two tailed *t* test). Whiskers are ± standard deviation.

After each experiment, gene expression was determined using qPCR (Figure 3) with TaqMan probes and analyzed by the 2^–Δ*CT*^ method using the TATA-box binding protein (TBP) as a housekeeping gene. In the “Differentiation only” experiment, the hPSC-CMs cultured on P(NDI2OD-T2) at day 19 showed no discernible morphological or gene expression differences compared to the control group when maturation was not allowed. Conversely, in the “Differentiation + maturation” experiment, hPSC-CMs on P(NDI2OD-T2) displayed enhanced expression of the CM maturation markers MYH6, MYH7, and NPPA, the calcium handling markers RYR2 and CACNA1C, the ion conduction markers SCN5A and HCN4, the energetics marker PPARGC1A, and the pacemaker marker TBX18, by day 41 compared to those on control substrates. Remarkably, when we transferred hPSC-CMs that were grown on a tissue culture plate to the P(NDI2OD-T2) substrate, the cells on day 41 consistently exhibited elevated expression levels of maturation markers at day 41 compared to those on control substrates (“Maturation only”). These results provide evidence that P(NDI2OD-T2) contributed to an increased expression of maturation markers following the CM differentiation phase.

### Effect of Light Exposure on the Behavior of hPSCs

**Figure 4:**
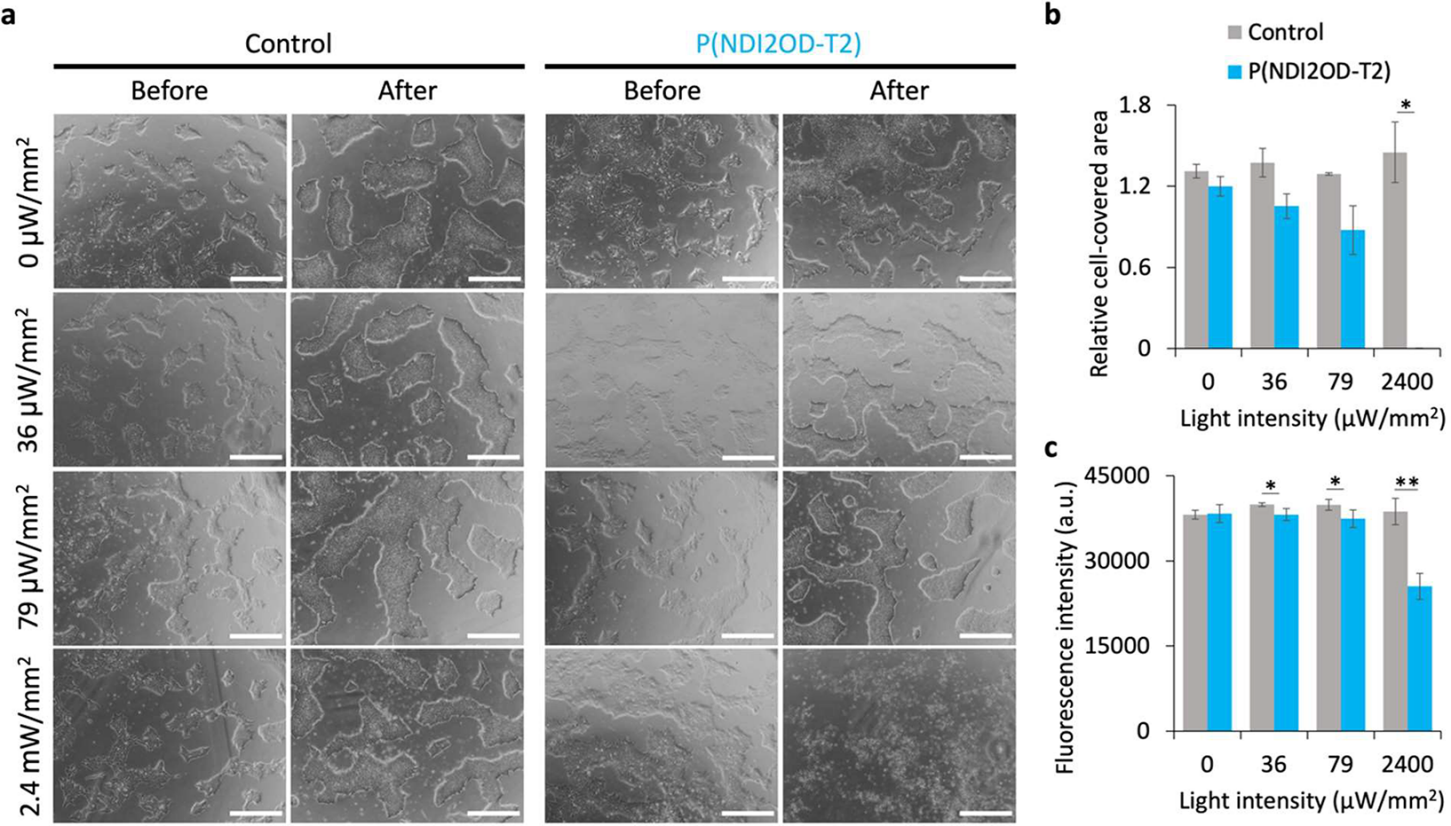
Exposingthe hPSCs to light on ITO and P(NDI2OD-T2) substrates. (A) Bright-field images of hPSCs cultures on control (ITO) and P(NDI2OD-T2) substrates before and after 24 h of illumination with 660 nm light pulses of 20 ms (at 1 Hz). The pulse intensity varied between 36 μW/mm^2^ and 2.4 mW/mm^2^. The scale bar is 200 μm. (B) Relative cell-covered area at different light intensities, defined as the cell-covered area after illumination relative to the cell-covered area before illumination. (C) Cell viability was measured using the fluorescence of alamarBlue after illumination. The cell-covered area was assessed from *n* = 3 wells for each condition and the cell viability was assessed with *n* = 6 corresponding to 3 independent wells per condition with two technical replicates. * *p* < 0.05, ** < 0.0001 (unpaired two-tailed *t* test). Whiskers are ± standard deviation.

Electrical stimulation has been successful in inducing CM maturation through prolonged days or weeks of electrical pacing in hPSC-CMs,^4,6,30−33^ while most optical pacing experiments relied on short-term light exposure.^12−15^ It is therefore crucial to establish a long-term light exposure protocol to optically pace hPSC-CMs on P(NDI2OD-T2) while preserving cell integrity. Our approach involved applying 660 nm light in pulses of 20 ms duration at a frequency of 1 Hz for 24 h, starting from d0 (after the cells had been on these substrates for 48 h), with light intensities ranging from 36 μW/mm^2^ to 2.4 mW/mm^2^. When exposed to 36 μW/mm^2^, the hPSCs on P(NDI2OD-T2) exhibited a normal cell attachment and colony morphology comparable to those of hPSCs on control substrates exposed to the same light intensity (Figure 4a). However, upon exposure to light intensities exceeding 36 μW/mm^2^, the cells on P(NDI2OD-T2) either detached or displayed a retracted colony edge, whereas those on control substrates remained intact even at the maximum light intensity conditions, i.e., 2.4 mW/mm^2^ (Figure 4a). The compromised hPSC colony morphology on P(NDI2OD-T2) was solely driven by light exposure, as hPSCs maintained in darkness (0 mW/mm^2^) on P(NDI2OD-T2) exhibited characteristics similar to those of the control.

Using these images, we quantified the cell-covered area on each substrate before and after 24-h illumination. The cell-covered area increased by around 20–40% in control substrates after exposure to all light intensities, as well as in the P(NDI2OD-T2) substrate but only when it was kept in the dark. In contrast, the cell coverage on P(NDI2OD-T2) at 36 μW/mm^2^ exhibited a modest increase of only about 5% (Figure 4b), suggesting an impaired cell proliferation even at this low light intensity. Increasing light intensities on P(NDI2OD-T2) led to a decrease in the cell-covered area, with a reduction of around 12% at 79 μW/mm^2^, and complete detachment of cells from the P(NDI2OD-T2) substrate at 2.4 mW/mm^2^ (Figure 4b). A similar trend was observed in the cell viability assessment on each substrate after 24 h illumination exposure (Figure 4c). The fluorescence conversion levels of alamarBlue remained comparable between the control substrates and the P(NDI2OD-T2) kept in darkness; however, a progressive reduction of fluorescence intensity was evident in P(NDI2OD-T2) relative to the control as the light intensity increased (Figure 4b). These results suggest that the observed cell damage on P(NDI2OD-T2) surfaces is a consequence of a light-induced process specific to P(NDI2OD-T2), rather than being attributed to the general phototoxicity of light or a low biocompatibility level of P(NDI2OD-T2) itself. We note that the film did not undergo chemical degradation upon light exposure (Figure S1). Based on this observation, we hypothesize that the film may generate species that are toxic to the cell membrane or detrimental to the adhesion of extracellular matrix proteins.

To evaluate whether P(NDI2OD-T2) can exert pacing control over hPSC-CMs, we increased the light intensity to 0.5 mW/mm^2^, but to ensure no cell death, we stimulated the cells in a transient regime. We exposed the hPSC-CMs at day 41 with 20 ms light pulses (at a frequency of 1 Hz for 30 s) for a few minutes. This setting aligns with the successful stimulation protocol used by Lodola et al.^14^ when factoring in the distance between the LED and the sample. The cells did not alter their beating frequency during or after light exposure under these conditions. We also performed the experiment with a single light pulse of 30 s, and the cells did not change the beating frequency (see Figure S2 for an exemplary trace). Note that light exposure at this intensity induced only a marginal change in the OCP of P(NDI2OD-T2) by +30 mV. Lower intensities, such as 30 μW/mm^2^, did not alter the OCP (Figure S3a). Additionally, light exposure at 30 μW/mm^2^ or 0.5 mW/mm^2^ did not cause any change in the temperature of the cell media close to the substrate (Figure S3b). In summary, long-term light exposure above 36 μW/mm^2^ produces toxic effects, and short-term stimulation does not affect the beating frequency of hPSC-CMs. Thus, we conclude that while P(NDI2OD-T2) enhances maturation simply as a substrate material, it is not efficient for light-based CM pacing.

### Why Does the P(NDI2OD-T2) Coating Enhance hPSC-CM Maturation?

**Figure 5:**
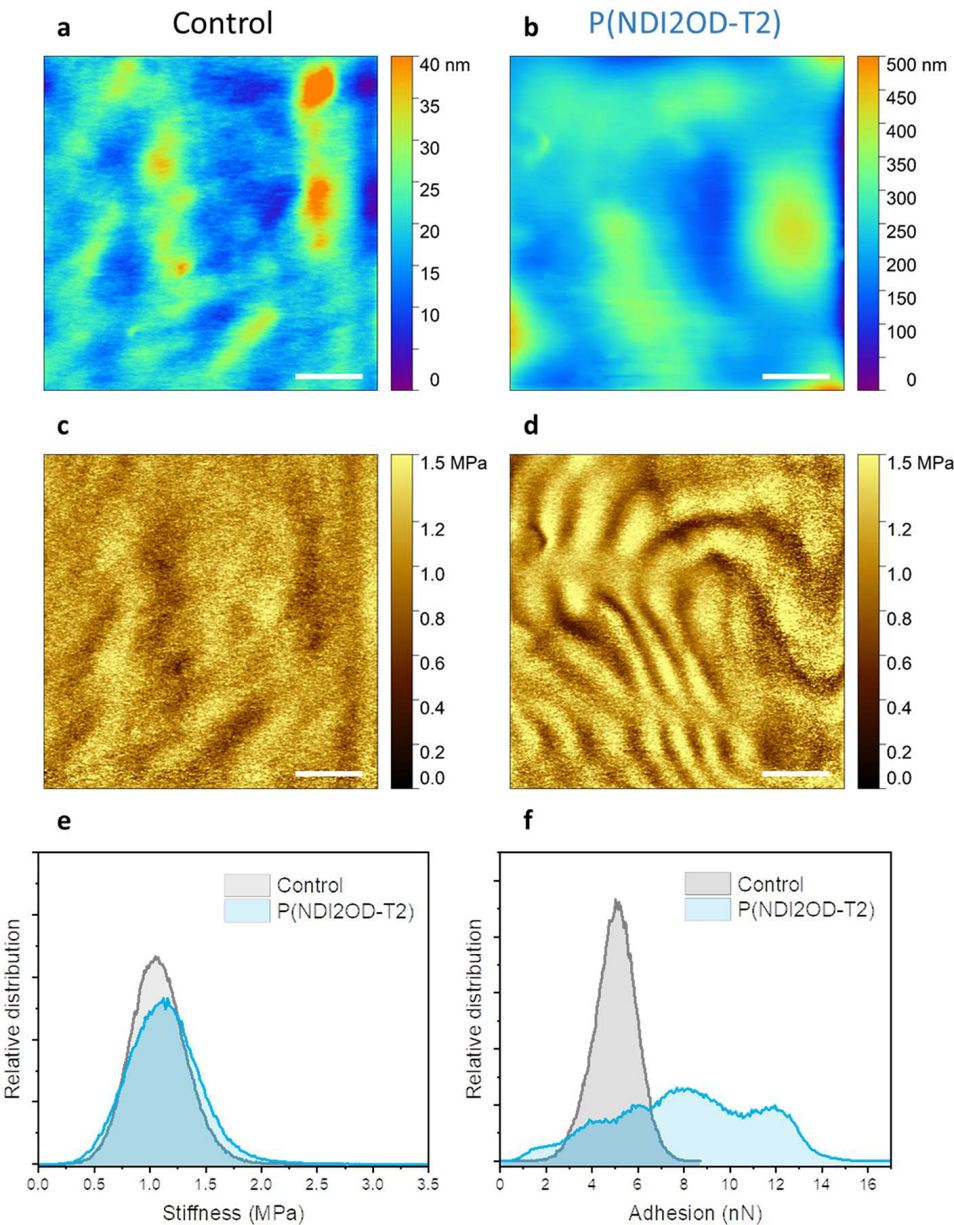
AFM topography images of Matrigel-coated on the (A) ITO substrate and (B) P(NDI2OD-T2) substrate immersed in cell media. Stiffness profiles were extracted from the locations probed in topography images for the Matrigel coating on the (C) ITO substrate and (D) P(NDI2OD-T2) substrate. The scale bar is 2 μm. The extracted distributions of (E) stiffness and (F) adhesion force between the AFM tip and Matrigel.

P(NDI2OD-T2) coating can change the surface properties of the substrate, potentially influencing the differentiation and maturation of hPSC-CMs, as evidenced by the qPCR results shown in Figure 2d. However, on top of the P(NDI2OD-T2) film (or ITO), we coated a layer of Matrigel. Matrigel, as an extracellular protein matrix, is essential for cell adhesion; without such coatings, the cells fail to attach to the substrate. We hypothesize that the physicochemical properties of P(NDI2OD-T2) affect Matrigel, thereby promoting the maturation of hPSC-CMs. To investigate this hypothesis, we first analyzed the surface properties of the Matrigel-coated substrates using atomic force microscopy (AFM). Comparing the AFM topography images, we observed that the Matrigel on ITO displayed much smaller peaks (Figure 5a) compared to that on P(NDI2OD-T2) (Figure 5b). On ITO, the highest topographic peak is 40 nm, while on P(NDI2OD-T2), peaks reach a height of 500 nm. Such larger features, or wrinkles, have been shown to enhance the alignment of CMs and promote maturation.^34^ Furthermore, the stiffness map of Matrigel on ITO appeared mostly homogeneous (Figure 5c), while Matrigel on P(NDI2OD-T2) exhibited more large-scale structures in the form of ridges (Figure 5d). The Matrigel displayed alternating soft and hard wrinkles on the n-type film, exhibiting an even greater structural heterogeneity. However, despite these differences, on average, the stiffness of both samples was similar, suggesting that the cells were exposed to surfaces with comparable stiffness on the macroscale (Figure 5e).

Interestingly, although the average stiffness of the Matrigel on ITO and P(NDI2OD-T2) was similar, the adhesion forces between the AFM tip and the Matrigel differed significantly on these substrates, as demonstrated in the adhesion profiles depicted in Figure 5f. On P(NDI2OD-T2), Matrigel displayed a heterogeneous adhesion profile compared to the control, which we attributed to the differences in the arrangement of the proteins on the substrate. Overall, the large features observed in topography images and the ridged stiffness map of Matrigel on P(NDI2OD-T2) indicated the presence of a structured, heterogeneous surface for the cells to adhere onto. This unique surface structure could potentially trigger hPSC-CMs to change their adhesion behavior, which leads to enhanced maturation. It has been observed that surface properties play a critical role in the strength of the focal adhesion and alignment of stem cell-derived CMs.^34^

**Figure 6:**
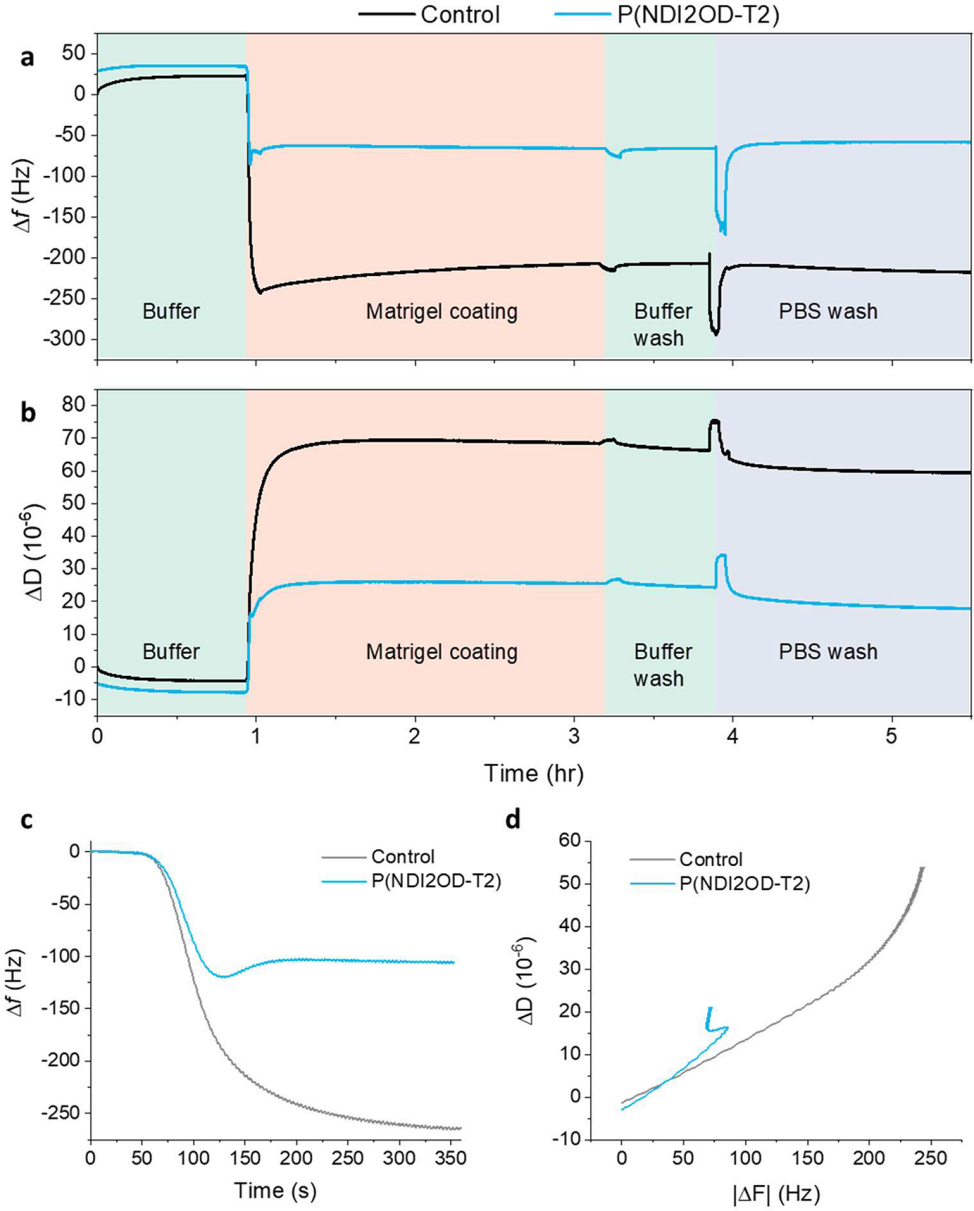
QCM-D (A) frequency (Δ*f*) and (B) dissipation (Δ*D*) shifts of the bare QCM-D sensor and the one coated with P(NDI2OD-T2). The chamber was at 37 °C. Once the sensors were stabilized in buffer (cell media), the Matrigel was introduced into the chamber, followed by a washing step to remove unbound Matrigel proteins. Subsequently, a high-rate flow of PBS was conducted to evaluate the stability of the Matrigel coating. The results are shown for the 3^rd^ overtone. (C) The change in Δf signals over time, (D) Δ*f* vs Δ*D* curve for bare and P(NDI2OD-T2)-coated sensors during Matrigel binding.

Next, we used a quartz crystal microbalance with dissipation monitoring (QCM-D) technique to understand the dynamics of Matrigel loading on P(NDI2OD-T2) compared to the control, and to quantify the amount of the Matrigel attaching on each surface and its viscoelastic properties. We compared the properties of a bare QCM-D sensor to a sensor coated with P(NDI2OD-T2) during the deposition of the Matrigel coating, followed by a washing step (Figure 6a, b). As the Matrigel was introduced into the chamber, we observed a decrease in frequency shifts accompanied by an increase in dissipation. Approximately 14.4 μg/cm^2^ of protein was bound to the surface of the bare sensor, while 7.37 μg/cm^2^ of protein adhered to the P(NDI2OD-T2) film. Following a washing step, minimal changes in the frequency and dissipation (∼3% for both sensors) confirmed the stability of the Matrigel layer on each surface. Even after an aggressive wash with phosphate-buffered saline (PBS), which applied significant shear force on the Matrigel layer, the Matrigel remained strongly bound to each surface, suggesting the stability of this layer. The binding of Matrigel on P(NDI2OD-T2) was notably faster than on the control (Figure 6c). Calculated from an exponential decay curve, the response time of Matrigel on control was 52.4 s, while on P(NDI2OD-T2), it was 20.7 s. We attribute the low amount of protein loading on P(NDI2OD-T2) to its hydrophobic nature (Figure S4). Consequently, the proteins could expand on P(NDI2OD-T2), resulting in a higher structural flexibility (Figure 6d). Such flexibility and softness (deduced from the higher dissipation values for the P(NDI2OD-T2)) may facilitate the formation of the features observed in AFM images, contributing to the heterogeneity in surface topography and stiffness. As the proteins had more flexibility, they settled into heterogeneous structures that formed a wrinkled surface. These topographies have been shown to enhance CM maturation by promoting better cell alignment and attachment.^35,36^

**Figure 7:**
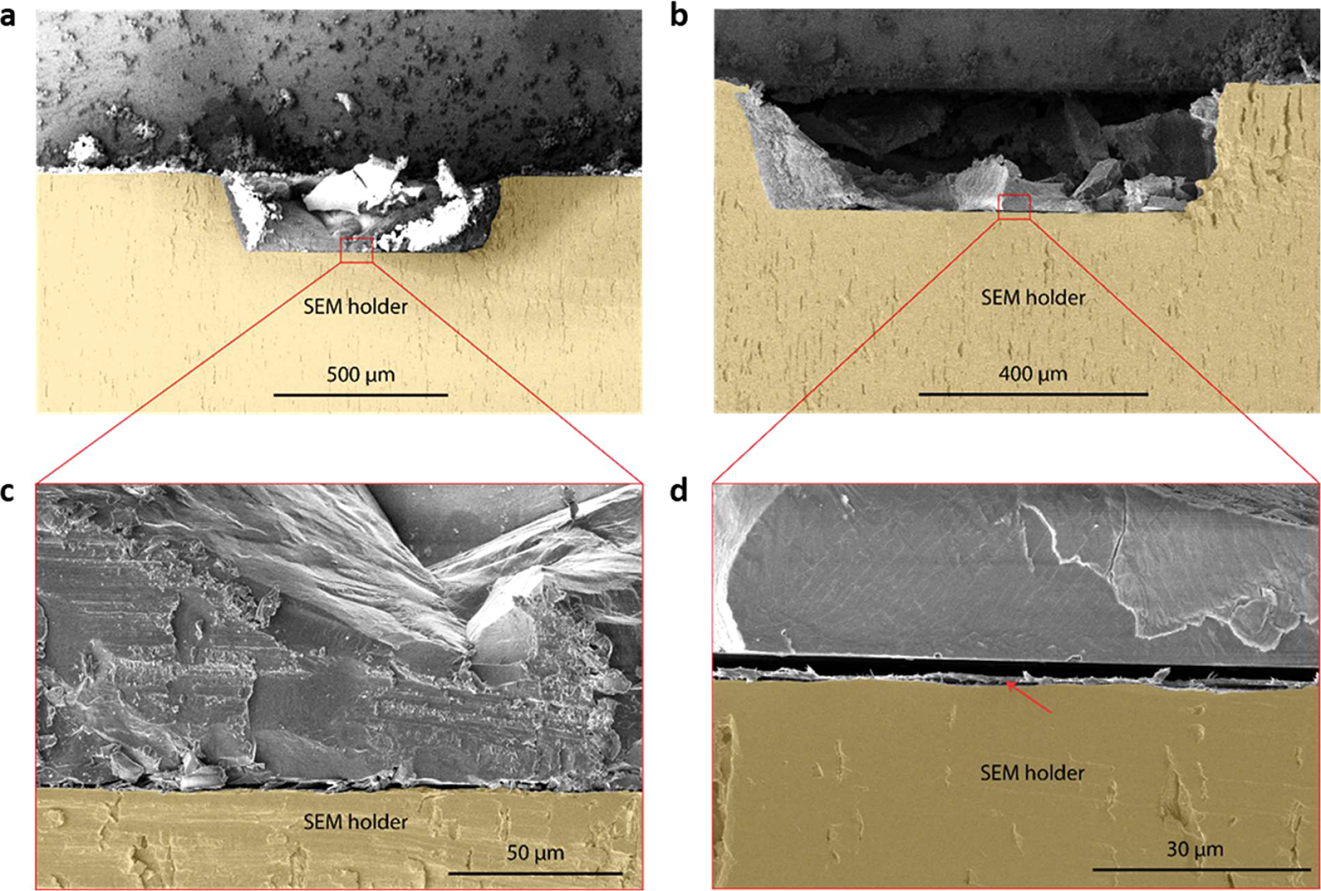
Cross-section scanning electron microscopy (SEM) images of Matrigel on a (A) bare SEM holder and (B) P(NDI2OD-T2)-coated holder. Zoomed images of the Matrigel interface with a (C) bare SEM holder and a (D) P(NDI2OD-T2)-coated holder.

Cross-sectional scanning electron microscopy (SEM) images provided further insights into the effect of the P(NDI2OD-T2) coating on the interactions between the Matrigel and the substrate. Samples were prepared by coating the Matrigel on bare SEM stubs and P(NDI2OD-T2)-coated stubs with cryogenic treatment that preserves the protein structure before freeze-fracturing for imaging. SEM images showed a significantly thinner Matrigel layer deposited on P(NDI2OD-T2)-coated samples (Figure 7b) compared to bare stubs (Figure 7a), in qualitative agreement with the QCM-D data (Figure 6). Magnified images revealed further differences at the Matrigel-substrate interface. On bare stubs (Figure 7c), Matrigel was intimately in contact with the surface, with little gap between them. In contrast, with the P(NDI2OD-T2) coating (Figure 7d), a noticeable gap was visible between Matrigel and the substrate. A thin layer above the substrate in Figure 7d, indicated by a red arrow, was presumed to be the P(NDI2OD-T2) coating. Notably, the Matrigel interface appeared smooth on the P(NDI2OD-T2) surface, unlike that of the heterogeneous surface on the bare sample. This smooth, separated interface suggests unfavorable interactions between Matrigel and P(NDI2OD-T2). The hydrophobic nature of P(NDI2OD-T2) hinders surface interactions of the Matrigel, inducing heterogeneity on the Matrigel surface interfacing with the cells. This could explain the large features observed in the AFM images of the Matrigel layer on P(NDI2OD-T2). We postulate that smaller amounts of protein arranged sparsely on the surface with minimal packing led to large peaks and valleys. The minimal packing allowed for a looser protein layer, which could explain the heterogeneity of the stiffness of Matrigel on P(NDI2OD-T2). Such a surface appears to positively affect the hPSC-CM maturation.

## Conclusions

In this work, we used the n-type semiconducting polymer P(NDI2OD-T2) as a light-sensitive material for the optical pacing of human pluripotent stem cell-derived cardiomyocytes (hPSC-CMs). Our first goal was to identify optimal light stimulation conditions that could effectively control hPSC-CM beating and maturation without causing phototoxicity for long-term cardiac pacing and maturation. We found that prolonged exposure to P(NDI2OD-T2)-mediated photostimulation at light intensities higher than 36 μW/mm^2^ resulted in cell damage. Although light could be safely applied via transient pulses, it did not induce changes in the hPSC-CM beating frequency. Only high light intensities caused changes in the open circuit potential of the film, and no light-induced change in the temperature of the medium was observed. These results indicate that P(NDI2OD-T2) may not be a suitable material for optical pacing applications.

Interestingly, we discovered that culturing hPSC-CMs on P(NDI2OD-T2)-coated substrates markedly enhanced their maturation compared to that of ITO . We found that P(NDI2OD-T2) influenced the loading and nanostructuring of the Matrigel coating in a way that promoted significant upregulation of key maturation markers related to contraction, calcium handling, metabolism, and conduction in hPSC-CMs. These results demonstrate that thin film coatings of semiconducting polymers can passively influence stem cell maturation by modulating the cell growth microenvironment. This conclusion highlights the importance of considering both active and passive effects when engineering photo- and electro-active biomaterials for tissue engineering or therapy. Future work should investigate the specific mechanisms underlying the toxic effects of P(NDI2OD-T2) under long term light exposure and determine whether similar cell-maturation-promoting effects can be observed with other conjugated polymers, particularly those with oligoether side chains that modulate the surface physicochemical properties.

## Materials and Methods

### Device Fabrication

ITO substrates (25 × 25 mm^2^) on glass (Xin Yan Technology) were cleaned with acetone and IPA in an ultrasonic bath and then dried. ITO substrates were activated with O_2_ plasma for 2 min at 150 W. P(NDI2OD-T2) (Ossila) dissolved in chlorobenzene was spin-coated on ITO at 1000 rpm for 45 s, then dried in air for 1 h. Glass wells (14 mm inner diameter and 17.5 mm height) were attached to the substrate using polydimethylsiloxane (PDMS).

### UV–Vis Absorbance Spectroscopy

Glass substrates were sequentially cleaned in acetone and isopropanol followed by oxygen plasma cleaning. P(NDI2OD-T2) was spin-coated on a glass or ITO substrate from a 5 mg/mL chlorobenzene solution. A Cary 5000 UV–vis-NIR Spectrophotometer was used to acquire the absorption spectrum of the P(NDI2OD-T2) film under dry conditions, with a 2 nm step.

### Light Stimulation

All light stimulation experiments were performed using a 660 nm LED (Thorlabs) except for stimulation in Figure S2, which was done using a 635 nm LED (Leica). Irradiance was measured using a Thorlabs PM100D power meter at the device location.

### Electrochemical Characterization

Cyclic voltammetry (CV) and open circuit potentiometry were performed using a BioLogic VSP-300 potentiostat. CV characterization was done from 0.2 to −0.8 V vs Ag/AgCl and using a platinum mesh as a counter electrode. All measurements were taken in E8 media (Sigma-Aldrich) with a scan rate of 100 mV/s.

### Matrigel Preparation and Coating

The hESC-qualified Matrigel (Corning) was thawed overnight on ice at 4 °C, diluted 1:20 with cold DMEM/F12 medium, and stored at 4 °C for up to 2 weeks. The coating was done by pouring Matrigel in tissue culture plates, ITO, and P(NDI2OD-T2) wells and incubating for 1 h in a humidified incubator at 37 °C.

### Human Pluripotent Stem Cell Culture and Light Exposure Experiments

The hESC (WA01) cell line was cultured based on the protocol outlined in Astro et al, 2022.^37^ The hESCs were cultured on vitronectin-coated tissue culture plates (Thermo Fisher Scientific) with Essential 8 Medium (E8, Thermo Fisher Scientific) within a humidified incubator set at 37 °C with 5% CO_2_. Passage of hESCs occurred approximately every 3 to 4 days, maintained at around 70% confluency using Accutase (STEMCELL Technologies). Additionally, E8 Medium included supplementation of 10 μM ROCK inhibitor Y-27632 (Sigma) for the initial 24 h of culture. To test the biocompatibility and phototoxicity of ITO and P(NDI2OD-T2), hESCs were cultured on Matrigel (Corning) coated ITO or P(NDI2OD-T2) wells and exposed to light stimulation 48 h after seeding. We used triplicates for each condition (control/P(NDI2OD-T2), differentiation only/differentiation+maturation/maturation only). The light stimulation consisted of light pulses of 20 ms at a frequency of 1 Hz for 24 h, with light intensities ranging from 36 μW/mm^2^ to 2.4 mW/mm^2^. We took pictures of the hPSC culture on the control substrate and P(NDI2OD-T2) before and after the light stimulation and quantified the cell-covered area using ImageJ software. We assessed the hPSC viability after 24 h of light exposure using the alamarBlue reagent (Thermofisher). Briefly, after the light exposure, cells were washed with PBS and incubated with alamarBlue diluted 1/10 in fresh E8 media at 37 °C with 5% CO_2_ in darkness for 4 h. Later, the supernatant of each sample was transferred to 3 wells of a 96-well plate, and fluorescence was read in a plate reader (TECAN) using a fluorescence excitation wavelength of 540–570 nm and emission at 580–610 nm. There were technical triplicates per sample and *n* = 3 samples per condition.

### Human Pluripotent Stem Cell Differentiation into Cardiomyocytes and hPSC-CM Splitting

The hESCs were differentiated to cardiomyocytes as described in Astro et al., 2023.^38^ Briefly, hESC were differentiated on Matrigel (Corning) coated tissue culture plates, ITO or P(NDI2OD-T2) wells, employing the PSC Cardiomyocyte Differentiation Kit (Thermo Fisher Scientific) following the manufacturer’s instructions. Passage of hPSC-CM differentiated on Matrigel-coated tissue culture plates occurred on differentiation day 19 using trypsin-EDTA (0.25%, Thermo Fisher Scientific). hPSC-CMs were seeded on Matrigel-coated ITO or P(NDI2OD-T2) wells using Cardiomyocyte Maintenance Medium (Thermo Fisher Scientific) supplemented with 10% KnockOut Serum Replacement (Thermo Fisher Scientific) and 10 μM ROCK inhibitor Y-27632 (Sigma) for the initial 48 h. Media change was done every other day using the Cardiomyocyte Maintenance Medium.

### Gene Expression Analysis

Extraction of total RNA was performed utilizing the MagMAX mirVana Total RNA Isolation Kit and the KingFisher Duo Prime system following the manufacturer’s guidelines. Subsequently, cDNA synthesis was carried out by employing the SuperScript VILO IV cDNA Synthesis Kit. Evaluation of gene expression was conducted through real-time qPCR using the QuantStudio 3 Real-Time PCR System along with TaqMan Fast Advanced Master Mix and 10 μM TaqMan Gene Expression Probes (Table S1). Normalization of individual gene expression was accomplished relative to that of TBP.

### Quantification and Statistical Analysis

The cell-covered area, cell viability, and gene expression data were analyzed using GraphPad Prism 5 for Windows. An unpaired two-tailed *t* test was used to compare the mean values of at least three replicates in each experiment. * *p* < 0.05, ** < 0.0001.

### Atomic Force Microscopy (AFM)

AFM was performed with a Veeco Dimension 3100 Scanning Probe System. We used a NCHV probe (Bruker) (Spring constant: 40 N/m). The substrates were fabricated as described in the Device Fabrication section but without glass wells. AFM measurements were done in RPMI media (Sigma-Aldrich) that was filtered with a 0.45 μm fiberglass filter. Gwyddion software was used for analysis and data post-treatment.

### Quartz Crystal Microbalance with Dissipation Monitoring (QCM-D)

Measurements were conducted using a Q-sense analyzer (QE401, Biolin Scientific) to monitor protein adsorption on the gold-coated quartz crystal in the presence and absence of the P(NDI2OD-T2) coating. The changes in frequency (Δ*f*) and dissipation (Δ*D*) were monitored at the third, fifth, seventh, ninth, and 11th harmonics. The QCM-D chamber was heated to 37 °C. The signals were first stabilized in buffer (E8 media, Sigma-Aldrich), and the Matrigel solution was then fed into the chamber at a flow rate of 100 μL/min. The sensor was incubated with the Matrigel for 2 h and then rinsed with the buffer at flow rate of 100 μL/min to remove loosely bound proteins, the fluidic pump was stopped and the signals were monitored over 35 min. The samples were rinsed in the chamber with 1× PBS at a flow rate of 579 μL/min which ensured a high shear force, and the signals were subsequently monitored for 1.5 h. The same protocol was carried out for both the bare sensor and the sensor coated with P(NDI2OD-T2). The data of the third, fifth, and seventh harmonics were fitted to a viscoelastic model (Voigt model) to obtain mass values.

### Scanning Electron Microscopy (SEM)

Gold-coated copper carriers of 3 mm outer dimater, 1.5 mm inner diameter, and 0.2 mm of depth were coated with P(NDI2OD-T2) by drop-casting from a chlorobenzene solution and were left dry for 1 h in air. Matrigel was drop-casted on bare and P(NDI2OD-T2)-coated carriers and left at 37 °C for 30 min, then washed with E8 media. To characterize the cross-section structure, the samples were plunge frozen in liquid ethane and the frozen samples were transferred into an EM VCM loading station (Leica Microsystems) where they were fixed into a special freeze fracture holder precooled inside EM VCM. These samples were immediately transferred via the Leica VCT500 shuttle under cryogenic temperature into Leica ACE900 where they were etched at −100 °C for 2 min and finally coated with 4 nm thick Platinium layer. The freeze fractured cross sections were imaged inside the Helios G4 equipped with Leica cryo-stage which was precooled below −140 °C. Several images were taken at low and high magnification ranging from 200× to 80 000×. All cryo-SEM images were taken under a cryogenic temperature below −140 °C.

### Contact Angle

The contact angle of a 5 μL water droplet was measured on ITO and P(NDI2OD-T2) surfaces using an optical tensiometer (Biolin Scientific). We measured 3 samples for each substrate type.

## Supporting Information Available

UV–vis spectra of the n-type film before and after media incubation and long-term light exposure, beating profile of cardiomyocytes before and after short-term light exposure, change in n-type film OCP and electrolyte temperature upon illumination, and the contact angle of the cell-culture substrates (PDF)

## References

[ref1] Dhahri W.; et al. In Vitro Matured Human Pluripotent Stem Cell–Derived Cardiomyocytes Form Grafts With Enhanced Structure and Function in Injured Hearts. Circulation 2022, 145, 1412–1426. 10.1161/CIRCULATIONAHA.121.053563. 35089805

[ref2] Sirenko O.; et al. Assessment of beating parameters in human induced pluripotent stem cells enables quantitative in vitro screening for cardiotoxicity. Toxicol. Appl. Pharmacol. 2013, 273, 500–507. 10.1016/j.taap.2013.09.017. 24095675 PMC3900303

[ref3] Zhang M.; D’Aniello C.; Verkerk A. O.; Wrobel E.; Frank S.; Ward-van Oostwaard D.; Piccini I.; Freund C.; Rao J.; Seebohm G.; et al. Recessive cardiac phenotypes in induced pluripotent stem cell models of Jervell and Lange-Nielsen syndrome: disease mechanisms and pharmacological rescue. Proc. Natl. Acad. Sci. U. S. A. 2014, 111, E5383–5392 10.1073/pnas.1419553111. 25453094 PMC4273331

[ref4] Gomez-Garcia M. J.; Quesnel E.; Al-attar R.; Laskary A. R.; Laflamme M. A. Maturation of human pluripotent stem cell derived cardiomyocytes in vitro and in vivo. Seminars in Cell & Developmental Biology 2021, 118, 163–171. 10.1016/j.semcdb.2021.05.022. 34053865

[ref5] Zhu R.; et al. Physical developmental cues for the maturation of human pluripotent stem cell-derived cardiomyocytes. Stem Cell Research & Therapy 2014, 5, 117 10.1186/scrt507. 25688759 PMC4396914

[ref6] Karbassi E.; et al. Cardiomyocyte maturation: advances in knowledge and implications for regenerative medicine. Nature Reviews Cardiology 2020, 17, 341–359. 10.1038/s41569-019-0331-x. 32015528 PMC7239749

[ref7] Berger H. J.; et al. Continual electric field stimulation preserves contractile function of adult ventricular myocytes in primary culture. Am. J. Physiol. 1994, 266, H341–349. 10.1152/ajpheart.1994.266.1.H341. 8304516

[ref8] Lim D.; et al. Bioreactor design and validation for manufacturing strategies in tissue engineering. Biodes Manuf 2022, 5, 43–63. 10.1007/s42242-021-00154-3. 35223131 PMC8870603

[ref9] Savva A.; et al. Photo-Chemical Stimulation of Neurons with Organic Semiconductors. Advanced Science 2023, 10, 2300473 10.1002/advs.202300473. 37661572 PMC10625067

[ref10] Francia S.; et al. P3ht-Graphene Device for the Restoration of Visual Properties in a Rat Model of Retinitis Pigmentosa. Advanced Materials Technologies 2023, 8, 2201467 10.1002/admt.202201467.

[ref11] Savchenko A.; et al. Graphene biointerfaces for optical stimulation of cells. Science Advances 2018, 4, eaat0351 10.1126/sciadv.aat0351. 29795786 PMC5959318

[ref12] Parameswaran R.; et al. Optical stimulation of cardiac cells with a polymer-supported silicon nanowire matrix. Proc. Natl. Acad. Sci. U. S. A. 2019, 116, 413–421. 10.1073/pnas.1816428115. 30538202 PMC6329945

[ref13] Gentemann L.; et al. Modulation of cardiomyocyte activity using pulsed laser irradiated gold nanoparticles. Biomed. Opt. Express 2017, 8, 177–192. 10.1364/BOE.8.000177. 28101410 PMC5231291

[ref14] Lodola F.; Vurro V.; Crasto S.; Di Pasquale E.; Lanzani G. Optical Pacing of Human-Induced Pluripotent Stem Cell-Derived Cardiomyocytes Mediated by a Conjugated Polymer Interface. Adv. Healthcare Mater. 2019, 8, 1900198 10.1002/adhm.201900198. 31066237

[ref15] Ronchi C.; Galli C.; Tullii G.; Marzuoli C.; Mazzola M.; Malferrari M.; Crasto S.; Rapino S.; Di Pasquale E.; Antognazza M. R. Nongenetic Optical Modulation of Pluripotent Stem Cells Derived Cardiomyocytes Function in the Red Spectral Range. Advanced Science 2024, 11, 2304303 10.1002/advs.202304303. 37948328 PMC10797444

[ref16] Paltrinieri T.; et al. Understanding Photocapacitive and Photofaradaic Processes in Organic Semiconductor Photoelectrodes for Optobioelectronics. Adv. Funct. Mater. 2021, 31, 2010116 10.1002/adfm.202010116.

[ref17] Đerek V.; Rand D.; Migliaccio L.; Hanein Y.; Głowacki E. D. Untangling Photofaradaic and Photocapacitive Effects in Organic Optoelectronic Stimulation Devices Frontiers in Bioengineering and Biotechnology 2020, 8, 10.3389/fbioe.2020.00284. PMC7180391 32363183

[ref18] Feiner R.; Dvir T. A ray of light for treating cardiac conduction disorders. Proc. Natl. Acad. Sci. U. S. A. 2019, 116, 347–349. 10.1073/pnas.1819948116. 30563854 PMC6329953

[ref19] Yang X.; Pabon L.; Murry C. E. Engineering adolescence: maturation of human pluripotent stem cell-derived cardiomyocytes. Circ. Res. 2014, 114, 511–523. 10.1161/CIRCRESAHA.114.300558. 24481842 PMC3955370

[ref20] Brixi S.; Melville O. A.; Mirka B.; He Y.; Hendsbee A. D.; Meng H.; Li Y.; Lessard B. H. Air and temperature sensitivity of n-type polymer materials to meet and exceed the standard of N2200. Sci. Rep. 2020, 10, 4014 10.1038/s41598-020-60812-x. 32132588 PMC7055259

[ref21] Giovannitti A.; et al. N-type organic electrochemical transistors with stability in water. Nat. Commun. 2016, 7, 13066 10.1038/ncomms13066. 27713414 PMC5059848

[ref22] Yan H.; et al. A high-mobility electron-transporting polymer for printed transistors. Nature 2009, 457, 679–686. 10.1038/nature07727. 19158674

[ref23] Gross Y. M.; Ludwigs S. P(NDI2OD-T2) revisited – Aggregation control as key for high performance n-type applications. Synth. Met. 2019, 253, 73–87. 10.1016/j.synthmet.2019.04.017.

[ref24] Ash C.; Dubec M.; Donne K.; Bashford T. Effect of wavelength and beam width on penetration in light-tissue interaction using computational methods. Lasers in Medical Science 2017, 32, 1909–1918. 10.1007/s10103-017-2317-4. 28900751 PMC5653719

[ref25] Chiaravalli G.; Manfredi G.; Sacco R.; Lanzani G. Photoelectrochemistry and Drift–Diffusion Simulations in a Polythiophene Film Interfaced with an Electrolyte. ACS Appl. Mater. Interfaces 2021, 13, 36595–36604. 10.1021/acsami.1c10158. 34310106 PMC8397247

[ref26] Mosconi E.; et al. Surface Polarization Drives Photoinduced Charge Separation at the P3HT/Water Interface. ACS Energy Lett. 2016, 1, 454–463. 10.1021/acsenergylett.6b00197.

[ref27] Druet V.; et al. A single n-type semiconducting polymer-based photo-electrochemical transistor. Nat. Commun. 2023, 14, 5481 10.1038/s41467-023-41313-7. 37673950 PMC10482932

[ref28] Jakešová M.; et al. Optoelectronic control of single cells using organic photocapacitors. Science Advances 2019, 5, eaav5265 10.1126/sciadv.aav5265. 30972364 PMC6450690

[ref29] Castillo E. A.; Lane K. V.; Pruitt B. L. Micromechanobiology: Focusing on the Cardiac Cell–Substrate Interface. Annu. Rev. Biomed. Eng. 2020, 22, 257–284. 10.1146/annurev-bioeng-092019-034950. 32501769

[ref30] Ronaldson-Bouchard K.; et al. Advanced maturation of human cardiac tissue grown from pluripotent stem cells. Nature 2018, 556, 239–243. 10.1038/s41586-018-0016-3. 29618819 PMC5895513

[ref31] Eng G.; Lee B. W.; Protas L.; Gagliardi M.; Brown K.; Kass R. S.; Keller G.; Robinson R. B.; Vunjak-Novakovic G. Autonomous beating rate adaptation in human stem cell-derived cardiomyocytes. Nat. Commun. 2016, 7, 10312 10.1038/ncomms10312. 26785135 PMC4735644

[ref32] Nunes S. S.; et al. Biowire: a platform for maturation of human pluripotent stem cell–derived cardiomyocytes. Nat. Methods 2013, 10, 781–787. 10.1038/nmeth.2524. 23793239 PMC4071061

[ref33] Zhao Y.; et al. A platform for generation of chamber-specific cardiac tissues and disease modeling. Cell 2019, 176, 913–927. e918 10.1016/j.cell.2018.11.042. 30686581 PMC6456036

[ref34] Ahn H.; et al. Hierarchical Topography with Tunable Micro- and Nanoarchitectonics for Highly Enhanced Cardiomyocyte Maturation via Multi-Scale Mechanotransduction. Adv. Healthcare Mater. 2023, 12, 2202371 10.1002/adhm.202202371. 36652539

[ref35] Shi H.; et al. Profiling the responsiveness of focal adhesions of human cardiomyocytes to extracellular dynamic nano-topography. Bioactive Materials 2022, 10, 367–377. 10.1016/j.bioactmat.2021.08.028. 34901553 PMC8636819

[ref36] Afzal J.; et al. Cardiac ultrastructure inspired matrix induces advanced metabolic and functional maturation of differentiated human cardiomyocytes. Cell Rep 2022, 40, 111146 10.1016/j.celrep.2022.111146. 35905711

[ref37] Astro V.; et al. Fine-tuned KDM1A alternative splicing regulates human cardiomyogenesis through an enzymatic-independent mechanism. iScience 2022, 25, 104665 10.1016/j.isci.2022.104665. 35856020 PMC9287196

[ref38] Astro V.; Ramirez-Calderon G.; Adamo A. Protocol to measure calcium spikes in cardiomyocytes obtained from human pluripotent stem cells using a ready-to-use media. STAR Protocols 2023, 4, 102252 10.1016/j.xpro.2023.102252. 37060558 PMC10140149

