# Supplementary material for: Enhancing the Maturation of Human Pluripotent Stem Cell-Derived Cardiomyocytes with an n-Type Organic Semiconductor Coating
Source: ACS Appl Mater Interfaces. 2024 Apr 15;16(49):66900–10. doi: 10.1021/acsami.3c18919 (PMC11647761; doi:10.1021/acsami.3c18919)
Supplement: Supplementary file 1 — am3c18919_si_001.pdf [file am3c18919_si_001.pdf]

Enhancing the maturation of human pluripotent stem cell-derived cardiomyocytes with an n-type organic semiconductor coating

Supplementary Information

Gustavo Ramirez-Calderon[1]‡, Abdulelah Saleh[2]‡, Tania Cecilia Hidalgo Castillo[2], Victor Druet[2], Bayan Almarhoon[2], Latifah Almulla[2], Antonio Adamo*[1], and Sahika Inal*[2]

[1]Laboratory of Stem Cells and Diseases, Biological and Environmental Science and Engineering Division, King Abdullah University of Science and Technology (KAUST), Thuwal 23955-6900, Saudi Arabia.

[2]Organic Bioelectronics Laboratory, Biological and Environmental Science and Engineering Division, KAUST, Thuwal 23955-6900, Saudi Arabia.

‡ These authors contributed equally.

Corresponding Authors

sahika.inal@kaust.edu.sa

antonio.adamo@kaust.edu.sa

KEYWORDS: conjugated polymer, n-type, stimulation, qPCR, organic bioelectronics, cardiomyocyte, pluripotent, stem cell.

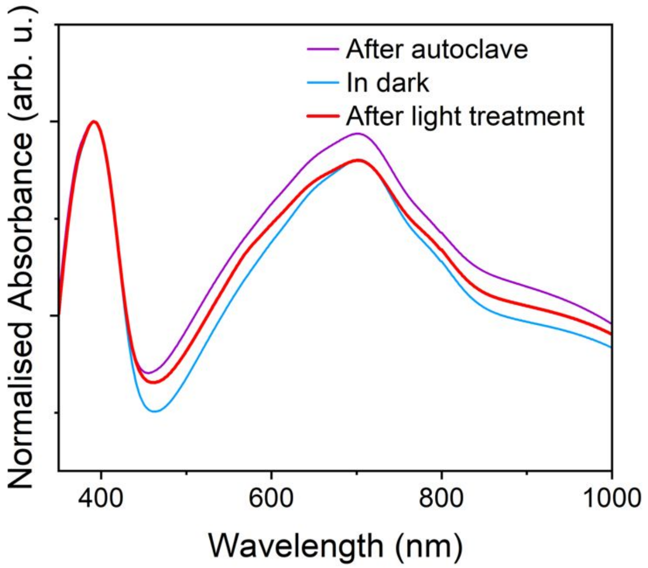

Figure S1. The UV-VIS spectrum of the film after autoclave, after being kept in cell media for 24 hours in the dark, and upon continuous stimulation with 660 nm light pulses of 20 ms at 1 Hz and at an intensity of 2.4 mW/mm^2.

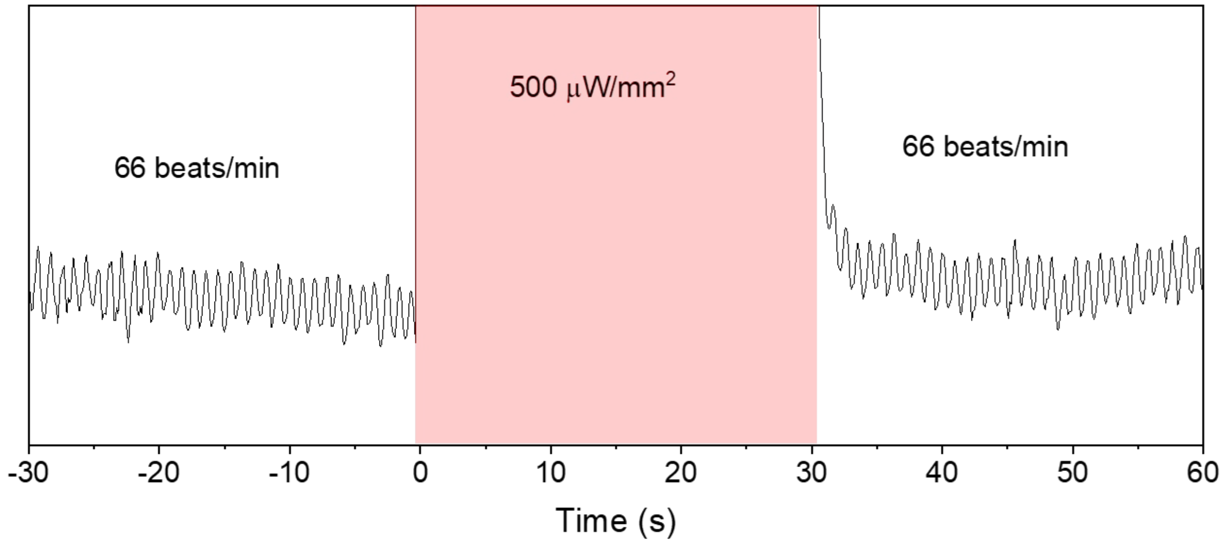

Figure S2. Beating profile of cardiomyocytes before and after stimulation with 500 μW/mm^2 660 nm LED for 30 s.

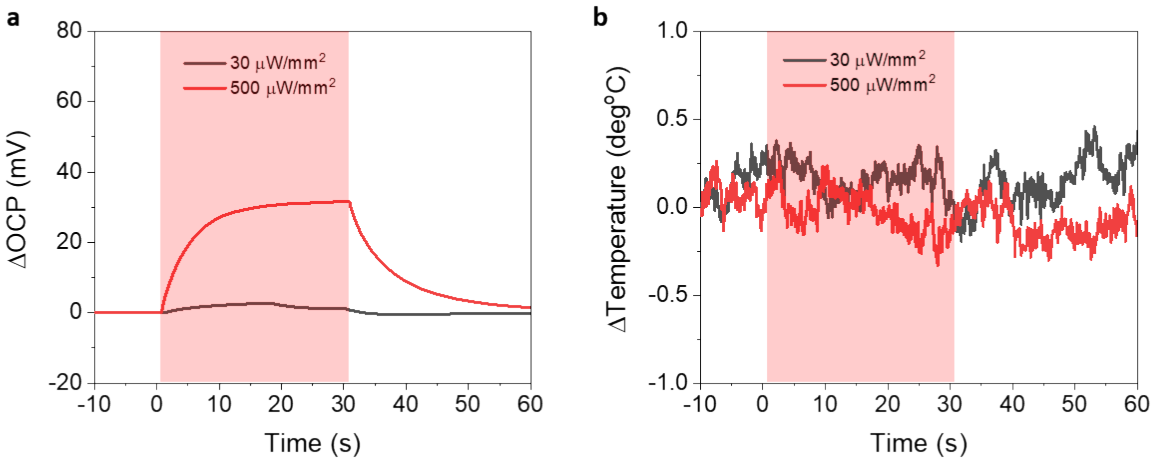

Figure S3. Change in **a)** OCP and **b)** temperature upon illumination of 30 μW/mm^2 and 500 μW/mm^2 of 660 nm LED.

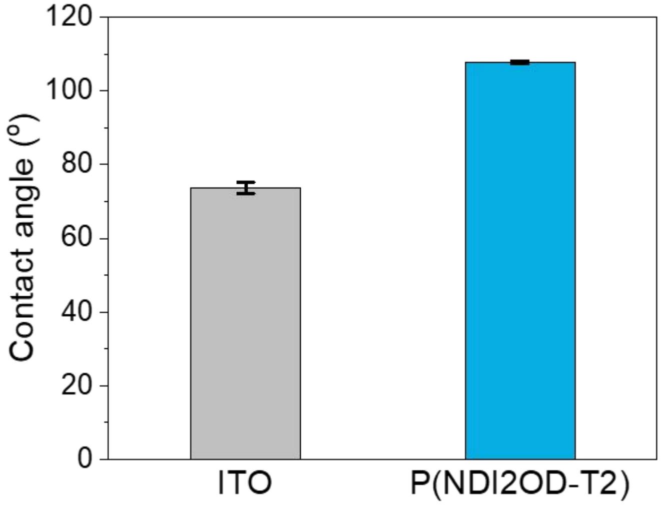

Figure S4. Contact angle of water on ITO and P(NDI2OD-T2) (*n=3*).

Table S1 TaqMan Gene Expression Probes.

Gene	Thermo Fisher Scientific Catalog number	Assay ID
ATP2A2	4331182	Hs00544877_m1
CACNA1C	4331182	Hs00167681_m1
CASQ2	4331182	Hs00154286_m1
GATA4	4331182	Hs00171403_m1
GJA1	4331182	Hs00748445_s1
HCN4	4331182	Hs00975492_m1
ISL1	4331182	Hs00158126_m1
KCNH2	4331182	Hs00542479_g1
KCNJ2	4331182	Hs00265315_m1
MAPK1	4331182	Hs01046830_m1
MEF2C	4331182	Hs00231149_m1
MSX2	4331182	Hs00741177_m1
MYH6	4331182	Hs01101425_m1
MYH7	4331182	Hs01110632_m1
NKX2.5	4331182	Hs00231763_m1
NPPA	4331182	Hs00383230_g1
NPPB	4331182	Hs00173590_m1
PPARGC1A	4331182	Hs00173304_m1
PRKAA1	4331182	Hs01562315_m1
PRKACA	4331182	Hs00427274_m1
RYR2	4331182	Hs00181461_m1
SCN5A	4331182	Hs00165693_m1
SHOX2	4331182	Hs00243203_m1
TBP	4331182	Hs00427620_m1
TBX18	4331182	Hs01385457_m1
TBX2	4331182	Hs00911929_m1
TBX5	4331182	Hs00361155_m1
TNNI3	4331182	Hs00165957_m1
TNNT2	4331182	Hs00943911_m1
